# Evolutionarily stable payoff matrix in hawk–dove games

**DOI:** 10.1186/s12862-024-02257-8

**Published:** 2024-05-20

**Authors:** Balázs Király, Tamás Varga, György Szabó, József Garay

**Affiliations:** 1 Institute of Technical Physics and Materials Science, HUN-REN Centre for Energy Research, Konkoly-Thege Miklós út 29–33., Budapest, H-1121 Hungary; 2 Bolyai Institute, University of Szeged, Aradi vértanúk tere 1., Szeged, H-6720 Hungary; 3 Institute of Evolution, HUN-REN Centre for Ecological Research, Konkoly-Thege Miklós út 29–33., Budapest, H-1121 Hungary

**Keywords:** Matrix game, Trait evolution, Hawk–dove game, Asymmetric interaction

## Abstract

**Background:**

Classical matrix game models aim to find the endpoint of behavioural evolution for a set of fixed possible interaction outcomes. Here, we introduce an evolutionary model in which not only the players’ strategies but also the payoff matrix evolves according to natural selection.

**Results:**

We start out from the hawk–dove matrix game and, in a way that is consistent with the monomorphic model setup of Maynard Smith and Price, introduce an evolving phenotypic trait that quantifies fighting ability and determines the probability of winning and the cost of losing escalated hawk–hawk fights. We define evolutionarily stable phenotypes as consisting of an evolutionarily stable strategy and an evolutionarily stable trait, which in turn describes a corresponding evolutionarily stable payoff matrix.

**Conclusions:**

We find that the maximal possible cost of escalating fights remains constant during evolution assuming a separation in the time scales of fast behavioural and slow trait selection, despite the fact that the final evolutionarily stable phenotype maximizes the payoff of hawk–hawk fights. Our results mirror the dual nature of Darwinian evolution whereby the criteria of evolutionary success, as well as the successful phenotypes themselves, are a product of natural selection.

**Supplementary Information:**

The online version contains supplementary material available at 10.1186/s12862-024-02257-8.

## Background

The birth of evolutionary game theory was motivated by a desire to understand why fights between males of the same species often do not result in serious injury [1]. Maynard Smith and Price described this phenomenon as “limited war”, which encapsulates two observations: a) Combat is often ritualized and involves no or limited physical contact [2–8]. b) If a fight does escalate, physical contact may still rarely result in injury due to, for example, the inefficiency of the weapons involved [9]. In the well-known hawk–dove game model of conflict, the cost of an escalated fight is just one of the payoff parameters that determine the evolutionarily stable probability of a player using the bellicose hawk strategy. This means that the classical hawk–dove game deals with how the evolutionary process of natural selection optimizes the frequency of ritualistic (dove strategy) and escalated, hazardous (hawk strategy) combat in a game theoretic sense. In other words, Maynard Smith and Price [1] focused on the first aspect of limited war. The hypothesis naturally arises that the evolutionary process minimizes the cost of escalated fights in a similar manner. The game theoretic discussion of this hypothesis requires an extension of the classical model framework that can actually account for the evolution and stability of said costs.

Maynard Smith and Price [1] defined evolutionarily stable strategies (ESS) as being impervious to invasion by mutant strategies when forming the overwhelming majority of a population. The basic question we ask in this paper is whether this notion of evolutionary stability can be extended to also include the evolution of the payoff matrix while following the assumptions of classical evolutionary matrix game theory (see [10]) as close as possible. This led us to reconsider what constitutes a phenotype. In the classical theory a phenotype is given by just a mixed strategy, a simple probabilistic decision-making rule that chooses from among a set of available pure strategies. Here we investigate the consequences of defining each phenotype through two independent attributes instead, namely one that describes behaviour (i.e., a mixed strategy) and one that quantifies a trait that determines possible interaction outcomes (i.e., the payoff matrix).

To our knowledge, this question has not previously been considered in the literature, although we have found some lines of research connected to it. The models that seem to have gained more traction use explicitly behaviour-dependent payoffs instead of independent traits to introduce a higher degree of phenotype-related variability. Some of them go so far as to intentionally abandon the idea of players acting according to a finite set of distinct behavioural patterns and with it the interpretative framework provided by matrix games in general (e.g., the continuous prisoner’s dilemma of Killingback and Doebeli [11, 12]). (Though both can be restored to these models, at least formally, by considering mixed strategies and strategy-dependent payoffs). Others [13–17] arrive at similarly non-linear payoffs as a consequence of assuming non-uniform interaction rates or durations between players following different strategies. The field of eco-evolutionary dynamics [18–23] treats the issue of feedback loops between fluctuations in population densities and behavioural decision-making by explicitly coupling the strategy frequencies to the achievable payoffs, too. Stochastic games modelling the benefits of sustained cooperation [24–27] also involve varying payoff values that dynamically depend on the strategic choices of the players. Our approach is much more similar to the one introduced by Akçay and Roughgarden [28], in which it is not the behaviour that is directly subject to mutation but rather the entries of the payoff matrix. We are surprised that this idea has not garnered more attention. One of the reasons why may be that the separation of the quick, “adaptive” evolution of behaviour (decision-making) and the slow, “paradigmatic” evolution of trait (e.g., physical strength and/or testosterone level), as mentioned by the authors themselves, evokes considering a new phenotype as a new pure strategy in a new, extended symmetric game as an alternative. In our setup, on the other hand, behaviour and trait are treated on an equal footing and evolve simultaneously, which naturally allows asymmetric interactions to take place between individuals having different traits.

One of the widely used and very successful methods of investigating trait evolution is adaptive dynamics [29–34]. Under adaptive dynamics, matrix games are not structurally stable, and thus indeterminate, rare special cases because of the linearity of their payoff functions [35, 36]. Nonetheless, we believe that our extended notion of evolutionarily stable phenotypes in matrix games, though narrower in scope, has the potential to open a new avenue of research into trait evolution that could complement adaptive dynamics by refining its description of certain situations. We hope that by combining linear and non-linear behaviours in the different coordinates of a two-dimensional phenotype space our model can close the apparent gap between the two theories.

## Methods

In keeping with trying to stay as close as possible to the evolutionary matrix game theory introduced by Maynard Smith and Price [1], we retain most of its well-known assumptions. We also consider an asexual population of a resident phenotype and arbitrary rare mutant phenotypes. The interactions are well-mixed, that is, each individual interacts with resident and mutant phenotypes with relative frequencies in line with the composition of the whole population. The interactions are pairwise and described by a matrix game; the fitness of a phenotype is given by its average payoff. We emphasize that customarily the payoff matrix is assumed to be fixed, the same regardless of which phenotypes participate in any given interaction. This is where we depart from standard evolutionary matrix game theory.

We start out from the well-known hawk-dove game [10]. In this symmetric game, players contest a resource of value *v* using one of two pure strategies called “hawk” and “dove”. “Doves” always avoid, “hawks” always escalate conflict, to the point of causing or receiving an injury of payoff value *c* if necessary. The game always has a clear winner who takes the whole resource and a clear loser who alone bears the cost of mutual escalation; when both players play the same pure strategy, the winner is chosen randomly. The well-known payoff matrix of the expectation value of the first player’s outcomes reads as

**Table:** 

	Hawk	Dove
Hawk	$\frac{1}{2}\left( v-c\right)$	*v*
Dove	0	$\frac{1}{2}v$

In the classical hawk–dove game, if $c>v$, then $\widetilde{\textbf{p}}=(\frac{v}{c},\frac{c-v}{c})$ is a mixed ESS. We emphasize that the coordinates of this classical ESS depend on the payoff of hawk–hawk interactions, as $\widetilde{\textbf{p}}$ depends on the cost of injury *c*.

We extend this framework to accommodate phenotypes with a second attribute beside the usual mixed strategy (i.e., the probabilities of the phenotype using its pure strategies in an interaction). Let us assume that the phenotypes differ in fighting ability – in other words, resource holding potential [37] influenced by, for example, their size, physical strength, skill, aggressiveness, etc. –, which determines both the probability of winning an escalated fight and the seriousness of injuries received in a lost fight. Let $m\in [0,1]$ denote this ability of a given individual and function $\pi :[0,1]\times [0,1]\rightarrow [0,1]$ give the probability of a hawk of ability $m_1$ winning an escalated fight against a hawk of ability $m_2$. This definition implies $\pi (m_1,m_2)=1-\pi (m_2,m_1)$, that is, that one hawk’s win is the other hawk’s loss. Moreover, let the function $c:[0,1]\times [0,1]\rightarrow [V,+\infty ]$ similarly determine the cost an $m_1$ hawk incurs when losing an escalated fight against an $m_2$ hawk. Taking our cue from the classical hawk–dove game, we also assume that the cost of an escalated fight is bigger than the resource value *v*, that is, $c>v$. Putting these pieces together, the expected payoff of the first hawk in an escalated fight is

1 $$\begin{aligned} a\left( m_1,m_2\right) =\pi \left( m_1,m_2\right) v-\left( 1-\pi \left( m_1,m_2\right) \right) c\left( m_1,m_2\right) . \end{aligned}$$

We assume that our two attributes, behaviour (the mixed strategies) and trait (now the fighting ability), are independent. This assumption has two important consequences: First, fighting ability only comes into play in escalated fights between hawks and has no bearing on interactions involving doves, so the corresponding entries of the original hawk–dove payoff are carried over without change into our model. Second, a mutation in trait introduces asymmetry into the familiar framework of the hawk–dove matrix game without stepping outside of the game theoretical conflict.

## Results

Now we are in a position to specify the payoff matrices that describe interactions within a monomorphic population of varying fighting ability. Still following in Maynard Smith’s footsteps, we assume that mutations occur rarely enough that they introduce at most one additional phenotype into the resident population at a time. The phenotypes are given by two attributes, mixed strategy and fighting ability. Formally, we represent the resident phenotype by $\left( \textbf{p}^*,m^* \right) \in S_2\times [0,1]$ and the mutant phenotype by $(\textbf{p},m)\in S_2\times [0,1]$, where $S_2=\{\textbf{q}=(q_1,q_2):q_1+q_2=1\text { and } q_1,q_2\ge 0\}$ is the standard 1-dimensional simplex. Let $\varepsilon \in (0,1)$ denote the relative frequency of the mutant phenotype in the population. The interaction outcomes are given by four payoff matrices: one for the symmetric conflict between two residents,

2 $$\begin{aligned} \textbf{A}:= \left( \begin{array}{cc} a(m^*,m^*) &{} v \\ 0 &{} \frac{v}{2} \end{array}\right) ; \end{aligned}$$

two for the asymmetric conflict between a resident and a mutant in trait,

$$\begin{aligned} \textbf{B}:= \left( \begin{array}{cc} a(m^*,m) &{} v \\ 0 &{} \frac{v}{2} \end{array}\right) \text { and } \textbf{C}:= \left( \begin{array}{cc} a(m,m^*) &{} v \\ 0 &{} \frac{v}{2} \end{array}\right) ; \end{aligned}$$

respectively; and one for the symmetric conflict between two mutants in trait,

$$\begin{aligned} \textbf{D}:= \left( \begin{array}{cc} a(m,m) &{} v \\ 0 &{} \frac{v}{2} \end{array}\right) . \end{aligned}$$

Now we are equipped to calculate the average fitness of the resident and mutant phenotypes as

$$\begin{aligned} W_{\textrm{R}}(\varepsilon )=(1-\varepsilon )\textbf{p}^*\textbf{A}\textbf{p}^*+\varepsilon \textbf{p}^*\textbf{B}\textbf{p} \end{aligned}$$

and

$$\begin{aligned} W_{\textrm{M}}(\varepsilon )=(1-\varepsilon )\textbf{p}\textbf{C}\textbf{p}^*+\varepsilon \textbf{p}\textbf{D}\textbf{p} \end{aligned}$$

respectively. From this, it follows that the average fitness of the whole population is

$$\begin{aligned} \overline{W}(\varepsilon )=(1-\varepsilon )W_{\textrm{R}}(\varepsilon )+\varepsilon W_{\textrm{M}}(\varepsilon ) \end{aligned}$$

Notice that the leading term of the mutant phenotype’s fitness is linear in the behavioural trait $\textbf{p}$ as is typical in matrix game theory and non-linear in the fighting ability trait *m* as is typical in adaptive dynamics theory.

Applying the verbal definition of Maynard Smith and Price [1], which identifies an evolutionarily stable strategy as *“a strategy such that, if most of the members of a population adopt it, there is no ‘mutant’ strategy that would give higher reproductive fitness”*, to small mutations in line with the perspectives of dynamical stability [38–42] and adaptive dynamics [34, 43], we arrive at the notion of monomorphic evolutionarily stable phenotypes:

$(\textbf{p}^*,m^* )\in S_2\times [0,1]$ is a monomorphic (*uniformly*) *evolutionarily stable phenotype* (ESP) with evolutionarily stable strategy (ESS) $\textbf{p}^*$ and evolutionarily stable payoff matrix (ESPM) $\textbf{A}$, if there is an $\varepsilon _0>0$ and a $\delta >0$ such that

3 $$\begin{aligned} W_{\textrm{R}}(\varepsilon )>W_{\textrm{M}}(\varepsilon ), \end{aligned}$$

whenever $\textbf{p}\ne \textbf{p}^*$, $0<\varepsilon <\varepsilon _0$, and $\left| m-m^*\right| <\delta$. (Just like for evolutionarily stable strategies [38, 39], the definition could be loosened to include non-uniformly evolutionarily stable phenotypes by allowing *m*- and $\textbf{p}$-dependent $\varepsilon _0(\textbf{p},m)$ bounds.)

The concept of evolutionary stability implicitly relies on the dynamic aspect of evolution (see, e.g., [40, 44]). By definition, fitness is the average growth rate of a phenotype. In this spirit, the monomorphic replicator dynamics for an arbitrary mutant phenotype $(\textbf{p},m )\ne (\textbf{p}^*,m^* )$ reads as

$$\begin{aligned} \dot{\varepsilon }=\varepsilon \left( W_{\textrm{M}}(\varepsilon )-\overline{W}(\varepsilon )\right) =\varepsilon (1-\varepsilon )\left( W_{\textrm{M}}(\varepsilon )-W_{\textrm{R}}(\varepsilon )\right) . \end{aligned}$$

Since Eq. (3) is equivalent to $W_{\textrm{M}}(\varepsilon )-W_{\textrm{R}}(\varepsilon )<0$, if $(\textbf{p}^*,m^* )$ is an ESP, then all other mutants become extinct according to the replicator dynamics, which indeed implies evolutionary stability.

To see that our ESP and the original ESS definitions are consistent, note that if $(\textbf{p}^*,m^*)$ is an ESP, then we have

- $$\textbf{p}^*\textbf{A}\textbf{p}^*>\textbf{p}\textbf{C}\textbf{p}^*$$
- if $$\textbf{p}^*\textbf{A}\textbf{p}^*=\textbf{p}\textbf{C}\textbf{p}^*$$, then $$\textbf{p}^*\textbf{B}\textbf{p}>\textbf{p}\textbf{D}\textbf{p}$$.

For all possible behavioural mutants $(\textbf{p},m^*)$ with $\textbf{p}\ne \textbf{p}^*$, $\textbf{A}=\textbf{B}=\textbf{C}=\textbf{D}$, and consequently the inequalities above reduce to the classical ESS definition from [1] for strategy $\textbf{p}^*$ with respect to the payoff matrix $\textbf{A}$ defined in Eq. (2).

When the two players are matched in fighting ability, that is $m_1=m_2=m$, the game becomes the usual hawk–dove game, and as a result has a mixed ESS at

4 $$\begin{aligned} \textbf{p}^*(m,m)=\left( \frac{v}{v-2a(m,m)},\frac{-2a(m,m)}{v-2a(m,m)}\right) \end{aligned}$$

as long as $a(m,m)<0$. With this in mind, we find (for details, see Lemma 1 in section SI.1 of Additional file 1) that

5 $$\begin{aligned} \begin{array}{cc} a(m^*,m^*)<0,\,\,\, &{}\partial _1a(m^*,m^*)=\partial _2a(m^*,m^*)=0,\\ \partial _{11}a(m^*,m^*)<0, &{}\partial _{22}a(m^*,m^*)>0 \end{array} \end{aligned}$$

give sufficient conditions for our modified hawk–dove game to have an interior, mixed ESP at $(\textbf{p}^*(m^*,m^*),m^*)$.

## Discussion

Comparing these conditions for evolutionary stability to the definition of evolutionary stability used in adaptive dynamics theory [43, 45] leads to some interesting analogies. We are interested in whether a mutant phenotype $(\textbf{p},m)$ can invade a resident $(\textbf{p}^*,m^*)$ phenotype, provided that $m\ne m^*$. It surely cannot if $\textbf{p}^*\textbf{A}\textbf{p}^*>\textbf{p}\textbf{C}\textbf{p}^*$, which is in line with the ESS component of the pairwise invasibility analysis presented in ([43], pg. 62 Question 1). This follows from criterion $\partial _{11} a(m^*,m^* )<0$, which essentially checks whether the resident phenotype’s fitness is locally maximal at $(\textbf{p}^* (m^*,m^*),m^*)$ by comparison to states characterized by an arbitrary different nearby trait *m*, and in this regard identifies $m^*$ as an ESS. (It turns out that the role of the behaviour $\textbf{p}$ is negligible in such comparisons, see Lemma 2 in section SI.2 of Additional file 1).

In the Background section, we set out to examine whether evolution minimizes the cost of escalated fights in a game theoretic sense. In order to do this, we have to first formalize what we mean by “in a game theoretic sense” in this context. Since we are interested in the final state of evolution, we consider homogeneous populations, in which all individuals have the same $(\textbf{p}(m,m),m)$. A member of this population, will incur a cost of *c*(*m*, *m*) if it plays hawk and loses against an opponent also willing to escalate. Since hawks are present in the population with a frequency of $p_1(m,m)$, the per game average of the maximal possible (worst-case scenario) cost of playing hawk against the population is given by the product $p_1(m,m)c(m,m)$. For any arbitrary $a(m_1,m_2)$ payoff not explicitly defined in the form prescribed by Eq. (1), we can assign a cost of injury function $c(m_1,m_2)=\frac{\pi (m_1,m_2)v-a(m_1,m_2)}{1-\pi (m_1,m_2)}$ that evidently satisfies Eq. (1) with the probability of winning function $\pi (m_1,m_2 )$. From Eqs. (1) and (4) it follows (see section SI.3 of Additional file 1) that

6 $$\begin{aligned} {p}^*_1(m,m)c(m,m)=v \end{aligned}$$

in a homogeneous population of fixed trait *m*, that is, the aforementioned worst-case, maximal possible cost is independent of *m* and equal to the value of the contested resource, while the probability of a player choosing to escalate a conflict $p_1^*(m,m)$ is inversely proportional to the potential cost of losing a fight *c*(*m*, *m*). In light of this result, limited war is not the result of an evolutionary minimization of the potential cost of conflict but rather a trade-off between different sources of risk, contrary to our seemingly natural hypothesis proposed in the Background section. If the evolution of traits is much slower than the evolution of behaviour, then the population effectively approaches the ESP $(\textbf{p}^*(m^*,m^* ),m^*)$ through a series of intermediate homogeneous stages $(\textbf{p}^*(m,m),m)$, and thus the more dangerous escalated fights become during this process due to evolution in trait, the less frequently they will occur due to evolution in behaviour, and vice versa.

Let us, finally, demonstrate through a simple example that our extended, one-trait hawk–dove game does indeed admit non-trivial, interior ESP solutions. Consider a trait-dependent hawk–hawk payoff function of the general expectation value form in Eq. (1), and specifically let each hawk’s winning probability be proportional to its relative fighting ability, so that

$$\begin{aligned} \pi (m_1,m_2)=\frac{m_1}{m_1+m_2} \end{aligned}$$

and each hawk’s trait-dependent injury cost function read as

7 $$\begin{aligned} c(m_1,m_2)=c_0+\alpha \left( m_1-\frac{1}{2}\right) ^2-\alpha \left( m_2-\frac{1}{2}\right) ^2=c_0+\alpha (m_1+m_2-1)(m_1-m_2). \end{aligned}$$

This is a plausible description of outcomes in an escalated conflict in which overall fighting ability is mainly determined either by a trait best applied in moderation – such as aggressiveness or courage, which may manifest as recklessness in excess and as timidity when lacking – or as a trade-off between two complementary traits – e.g., strength and quickness, the abilities to deliver and evade a decisive blow – with respect to the risk and extent of possible injury. Having a higher fighting ability than the opposing player ($m_1>m_2$) in this instance only lowers the cost of injury ($c(m_1,m_2 )<c_0$) as long as the total fighting ability of the two players does not exceed a threshold, that is, while $m_1+m_2<1$, and increases it otherwise.

**Fig. 1:**
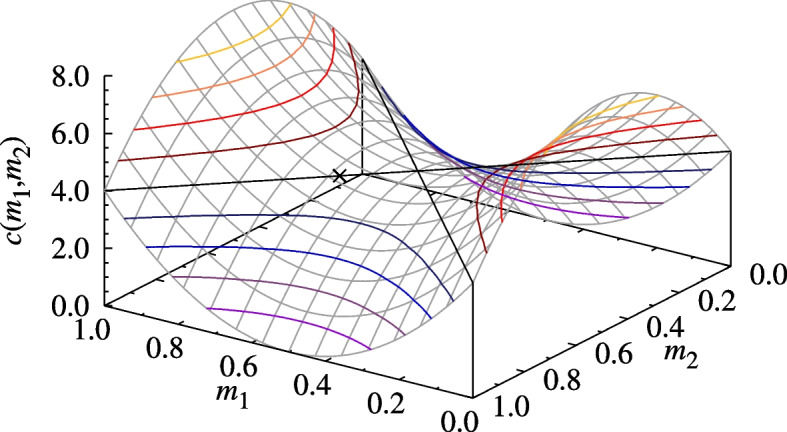
Surface plot of the injury cost function $c(m_1,m_2)$ of escalated hawk–hawk fights defined by the example given in Eq. (7) with parameter values $v=1$, $c_0=4$, and $\alpha =16$. $\mathbf \times$ shows the injury cost at the ESP Eq. (8)

**Fig. 2:**
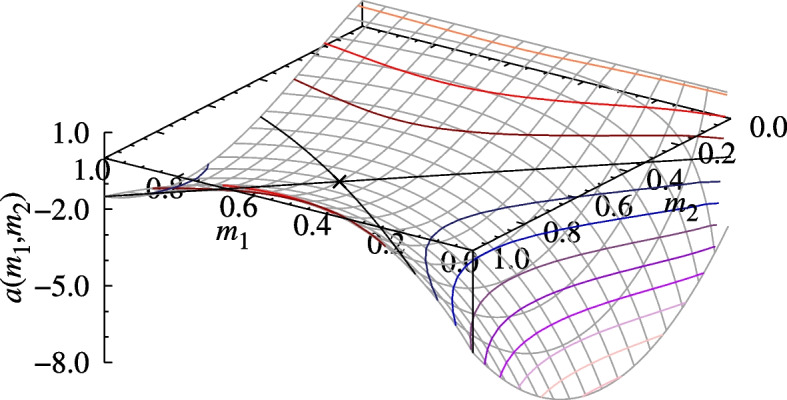
The example hawk–hawk interaction payoff function $a(m_1,m_2)$ ($v=1$, $c_0=4$, and $\alpha =16$) and its evaluation ($\mathbf \times$) at the ESP given in Eq. (8)

**Fig. 3:**
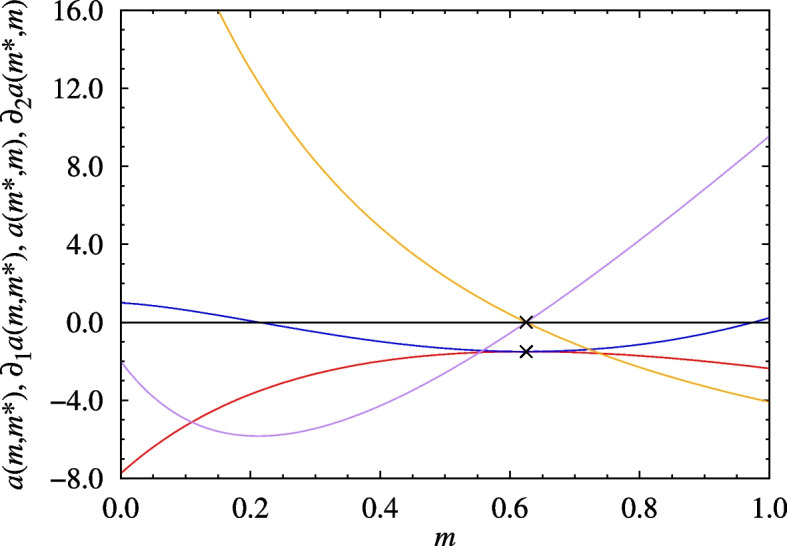
The cross sections of the example $a(m_1,m_2)$ payoff function when $v=1$, $c_0=4$, and $\alpha =16$ holding $m_1=m^*=\frac{5}{8}$ (blue line) and $m_2=m^*=\frac{5}{8}$ (red line) and their first derivatives (purple and orange lines, respectively). The $\mathbf {\times }$’s demonstrate that the ESP conditions of Eq. (5) are satisfied at $m^*=\frac{5}{8}$

It is easy to check that when the value of the resource is $v=1$ and the injury parameters are set to $c_0=4$ and $\alpha =16$, then the phenotype characterized by

8 $$\begin{aligned} m^*=\frac{5}{8},\qquad \textbf{A}= \left( \begin{array}{cc} \frac{v-c_0}{2} &{} v \\ 0 &{} \frac{v}{2} \end{array}\right) ,\qquad \textbf{p}^*(m^*,m^*)=\left( \frac{1}{4},\frac{3}{4}\right) \end{aligned}$$

satisfies the conditions for being an ESP. The injury cost, the hawk–hawk payoff functions, and its relevant cross-sections and partial derivatives with $m_1=m^*$ and $m_2=m^*$ are shown in Figs. 1, 2 and 3, respectively. Notice that the value of $a(m^*,m^*)$, the ESPM $\textbf{A}$, and as a result even $\textbf{p}^* (m^*,m^*)$, are not unique to $(m^*,m^*)$ in the sense that they are also taken for other $(m_1,m_2 )$ pairs (see the contour lines crossing at $(m^*,m^*)$ in Fig. 2.), though the ESP conditions are only satisfied at $(m^*,m^*)$. In particular, both *a*(*m*, *m*), *c*(*m*, *m*), and $\textbf{p}^* (m,m)$ are constant over all homogeneous states, which means that this example does not exhibit an evolution of limited war. This is not true in general. We illustrate this point in section SI.4 of Additional file 1 with two further examples, which locally maximize and minimize *c*(*m*, *m*), respectively.

## Conclusions

In this article, we propose an extension of the framework of matrix games and evolutionarily stable strategies towards an integrated description of the evolution of trait-dependent behaviour. We started out from the hawk–dove game, and focused on the outcomes of escalated hawk–hawk fights, as these are the only interactions in the original game where the fighting ability of the participants would determine the actual chance of winning and seriousness of injury. We took this consideration into account by considering hawk–hawk interactions as two-person, non-linear, symmetric subgames whose strategies are the trait. The resulting behavioural matrix game is asymmetric when played by phenotypes having different traits. We applied the verbal definition of evolutionary stability to the selection situation in which both behaviour and trait are subject to rare mutations, and demonstrated that the resulting definition of evolutionarily stable phenotypes is mathematically sound by providing sufficient conditions for their existence and a non-trivial example. Since an evolutionarily stable phenotype is an endpoint of the evolutionary process both in behaviour and in trait, the trait-dependent payoff matrix at an evolutionarily stable phenotype can also be thought of as an evolutionarily stable payoff matrix with respect to mutations in trait.

Our result is somewhat paradoxical: On the one hand, we found that the payoff of hawk–hawk fights (see the inequalities in Eq. (5)) is maximized by evolutionarily stable phenotypes (cf. [46]). At the bottom of this observation lies the fact that the appearance of a mutation in the fighting ability (*m*) introduces asymmetric interactions into the population (see section SI.1 of Additional file 1), which when taken into account together, provide the highest payoff to the evolutionarily stable phenotype’s strategy, $\textbf{p}^*(m^*,m^*)$. On the other hand, however, if we are only interested in whether evolution minimizes the cost of escalated fights in a game theoretic sense, we find that the maximal possible cost of escalating remains constant as evolution drives the population through a series of homogeneous states (see Eq. (6) and section SI.3 of Additional file 1). This is made possible by natural selection simultaneously changing the players’ strategy $\textbf{p}(m,m)$ and the payoff of hawk–hawk fights *a*(*m*, *m*). We think that the existence of such a game theoretic “conservation law” for the matrix games considered here is surprising. It also raises the question: Is there a similar “conservation law” for non-matrix games, whose payoffs depend non-linearly on the players’ strategy choices? It seems sensible that it is not worth fighting when its maximal possible cost is higher than the *v* value of the contested resource. Whether the maximal possible cost actually reaches the value of the resource during natural selection is an open question.

Our result that the payoff of hawk–hawk fights (see the inequalities in Eq. (5)), is maximized by evolutionarily stable phenotypes also establishes a connection between our matrix game and adaptive dynamics [35, 36, 47, 48] in the sense that our evolutionarily stable phenotype definition reproduces the standard adaptive dynamics definition of evolutionary stability for the trait (e.g., [43, 45]). From the point of view of adaptive dynamics theory “evolutionary matrix games are fundamentally degenerate” and structurally unstable due to their linearity [35, 36], an issue that persists for higher-dimensional traits, too [33, 34]. From this perspective, our model overlaps the fundamental assumptions of both matrix game theory and adaptive dynamics theory, as its fitness function is linear in its behavioural trait dimension and non-linear in its fighting ability trait dimension, and as a result provides a more refined description than the pure applications of either framework could.

Finally, we also note that our definition of evolutionarily stable phenotypes can be easily generalized to matrix games with any number of trait-dependent payoff entries. We also expect the lemma regarding the sufficient conditions for the existence of evolutionarily stable phenotypes to be extendable to these general cases. In a future study, we will explore how the local stability of the corresponding replicator dynamics follows from our definition.

### Supplementary Information

Additional file 1. Supplementary Information: The proofs of two lemmas regarding conditions for the existence of a symmetric ESP and invasibility in our generalized hawk–dove game and a comment highlighting the fact that the payoff and cost of hawk–hawk interactions can be both locally maximal or minimal in a symmetric ESP.

## Data Availability

No datasets were generated or analysed during the current study.

## Abbreviations

ESS: Evolutionarily stable strategy
ESP: Evolutionarily stable phenotype
ESPM: Evolutionarily stable payoff matrix
