# Supplementary material for: Evolutionarily stable payoff matrix in hawk–dove games
Source: BMC Ecol Evol. 2024 May 20;24:65. doi: 10.1186/s12862-024-02257-8 (PMC11107024; doi:10.1186/s12862-024-02257-8)
Supplement: Supplementary file 1 — Additional file 1. Supplementary Information: The proofs of two lemmas regarding conditions for the existence of a symmetric ESP and invasibility in our generalized hawk–dove game and a comment highlighting the fact that the payoff and cost of hawk–hawk interactions can be both locally maximal or minimal in a symmetric ESP. [file 12862_2024_2257_MOESM1_ESM.pdf]

Supplementary Information

to *Evolutionarily stable payoff matrix in hawk–dove games*
by Balázs Király, Tamás Varga, György Szabó, and József Garay

SI.1

Lemma 1. (Condition for symmetric ESP) *Let $(p_1^*, 1-p_1^*) = \mathbf{p}^*$ be an ESS with respect to the payoff matrix $\mathbf{A}$ defined in Eq. (2) of the main text. If*

$$\partial_1 a(m^*, m^*) = \partial_2 a(m^*, m^*) = 0$$

and

$$\partial_{11} a(m^*, m^*) < 0, \qquad \partial_{22} a(m^*, m^*) > 0,$$

then $(\mathbf{p}^*, m^*)$ is an ESP with respect to the hawk–dove game defined by the payoff function $a(m_1, m_2)$.

Proof. Using notation introduced in the main text, $(\mathbf{p}^*, m^*)$ is an ESP if there exist an $\varepsilon_0 > 0$ and a $\delta > 0$ such that Eq. (3), that is

$$(1-\varepsilon)\mathbf{p}^*\mathbf{A}\mathbf{p}^* + \varepsilon\mathbf{p}^*\mathbf{B}\mathbf{q} > (1-\varepsilon)\mathbf{q}\mathbf{C}\mathbf{p}^* + \varepsilon\mathbf{q}\mathbf{D}\mathbf{q},$$

holds whenever $\mathbf{q} \neq \mathbf{p}^*$, $|m - m^*| < \delta$ and $0 < \varepsilon < \varepsilon_0$.

Since $\mathbf{p}^*$ is an ESS with respect to $\mathbf{A}$, there is an ε^* such that

$$(1-\varepsilon)\mathbf{p}^*\mathbf{A}\mathbf{p}^* + \varepsilon\mathbf{p}^*\mathbf{A}\mathbf{q} > (1-\varepsilon)\mathbf{q}\mathbf{A}\mathbf{p}^* + \varepsilon\mathbf{q}\mathbf{A}\mathbf{q}$$

is true for every $\mathbf{q} \neq \mathbf{p}^*$ and $0 < \varepsilon < \varepsilon^*$ [cf. 1]. Therefore, if

$$(1-\varepsilon)\mathbf{p}^*\mathbf{A}\mathbf{p}^* + \varepsilon\mathbf{p}^*\mathbf{B}\mathbf{q} \geq (1-\varepsilon)\mathbf{p}^*\mathbf{A}\mathbf{p}^* + \varepsilon\mathbf{p}^*\mathbf{A}\mathbf{q}, \tag{SI.1}$$

and

$$(1-\varepsilon)\mathbf{q}\mathbf{A}\mathbf{p}^* + \varepsilon\mathbf{q}\mathbf{A}\mathbf{q} \geq (1-\varepsilon)\mathbf{q}\mathbf{C}\mathbf{p}^* + \varepsilon\mathbf{q}\mathbf{D}\mathbf{q} \tag{SI.2}$$

hold for any $0 < \varepsilon < \varepsilon_0$ with some appropriate $\varepsilon_0 \in (0, \varepsilon^*]$ in a neighbourhood of m^*, we are done.

Since ε is positive, inequality (SI.1) is equivalent to

$$\mathbf{p}^*\mathbf{B}\mathbf{q} \geq \mathbf{p}^*\mathbf{A}\mathbf{q},$$

which is satisfied if $a(m^*, m)$ has a local minimum at $m = m^*$. This occurs if

$$\partial_2 a(m^*, m^*) = 0 \qquad \text{and} \qquad \partial_{22} a(m^*, m^*) > 0.$$

Inequality (SI.2) can be rearranged as follows:

$$(1-\varepsilon)\mathbf{q}[\mathbf{A}-\mathbf{C}]\mathbf{p}^* + \varepsilon\mathbf{q}[\mathbf{A}-\mathbf{D}]\mathbf{q} \geq 0.$$

Performing the multiplications yields

$$(1-\varepsilon)q_1p_1^*[a(m^*,m^*)-a(m,m^*)]+\varepsilon q_1^2[a(m^*,m^*)-a(m,m)]\geq 0,$$

where $(q_1, 1-q_1) = \mathbf{q}$. This inequality is true if $q_1 = 0$ or the left-hand side has a strict minimum at $m = m^*$. The latter holds if $q_1 \neq 0$ and its first-order derivative with respect to m is 0 and its second order derivative with respect to m is positive at $m = m^*$, that is, if

$$-(1-\varepsilon)p_1^*\partial_1 a(m^*,m^*)-\varepsilon q_1[\partial_1 a(m^*,m^*)+\partial_2 a(m^*,m^*)]=0$$

and

$$-(1-\varepsilon)p_1^*\partial_{11} a(m^*,m^*)-\varepsilon q_1[\partial_{11} a(m^*,m^*)+2\partial_{12} a(m^*,m^*)+\partial_{22} a(m^*,m^*)]>0.$$

The first condition is fulfilled if $\partial_1 a(m^*,m^*) = \partial_2 a(m^*,m^*) = 0$, while the inequality is equivalent to

$$\partial_{11} a(m^*,m^*) < \frac{-\varepsilon q_1}{(1-\varepsilon)p_1^*}[\partial_{11} a(m^*,m^*)+2\partial_{12} a(m^*,m^*)+\partial_{22} a(m^*,m^*)].$$

Since the right-hand side tends to 0 as $\varepsilon \to 0$, the inequality holds if $\partial_{11} a(m^*,m^*) < 0$ and $0 < \varepsilon < \varepsilon_0$ for a small enough ε_0.

□

SI.2

Lemma 2. (Invasibility) *If $a(m,m^*)$ has a strict local maximum at $m = m^*$, in particular, if*

$$\partial_1 a(m^*,m^*) = 0 \qquad \text{and} \qquad \partial_{11} a(m^*,m^*) < 0, \quad \text{then} \tag{SI.3}$$

$\mathbf{p}^*\mathbf{A}\mathbf{p}^* > \mathbf{q}\mathbf{C}\mathbf{p}^*$ *for any* $\mathbf{q}$ *distinct from* $\mathbf{e}_2 = (0,1)$ *and* $m \neq m^*$ *close enough to* m^*.

Proof. Assume that $m \neq m^*$. We first show that

$$\mathbf{p}^*\mathbf{A}\mathbf{p}^* > \mathbf{e}_1\mathbf{C}\mathbf{p}^*. \tag{SI.4}$$

Performing the multiplications, the inequality takes the following shape

$$\frac{1}{2}\left(2a(m^*,m^*)(p_1^*)^2+v-(p_1^*)^2v\right) > a(m,m^*)p_1^*+(1-p_1^*)v.$$

Since $p_1^* = v/\big(v-2a(m^*,m^*)\big)$ (see Eq. (4) in the main text), the left-hand side is

$$\frac{v}{2}\,\frac{-2a(m^*,m^*)}{v-2a(m^*,m^*)},$$

and the right-hand side is

$$v \, \frac{a(m,m^*) - 2a(m^*,m^*)}{v - 2a(m^*,m^*)}.$$

We easily infer that inequality (SI.4) holds if

$$a(m^*,m^*) - a(m,m^*) > 0,$$

which follows from condition (SI.3).

On the other hand, since $\mathbf{p}^*$ is an ESS with respect to $\mathbf{A}$, it follows that $\mathbf{p}^*\mathbf{A}\mathbf{p}^* = \mathbf{e}_2\mathbf{A}\mathbf{p}^*$, and it is easy to check that $\mathbf{e}_2\mathbf{A}\mathbf{p}^* = \mathbf{e}_2\mathbf{C}\mathbf{p}^*$. Therefore, $\mathbf{p}^*\mathbf{A}\mathbf{p}^* = \mathbf{e}_2\mathbf{C}\mathbf{p}^*$. Hence, for $\mathbf{q} = q_1\mathbf{e}_1 + q_2\mathbf{e}_2$ with $q_1 > 0$, we immediately get

$$\mathbf{p}^*\mathbf{A}\mathbf{p}^* = \mathbf{q}\mathbf{A}\mathbf{p}^* = q_1\mathbf{e}_1\mathbf{A}\mathbf{p}^* + q_2\mathbf{e}_2\mathbf{A}\mathbf{p}^* > q_1\mathbf{e}_1\mathbf{C}\mathbf{p}^* + q_2\mathbf{e}_2\mathbf{C}\mathbf{p}^* = \mathbf{q}\mathbf{C}\mathbf{p}^*.$$

□

SI.3

An observation on the conservation of $p_1^*(m,m)c(m,m)$

If the payoff matrix is

$$\begin{pmatrix} a(m_1,m_2) & v \\ 0 & v/2 \end{pmatrix},$$

then

$$a(m_1,m_2) = \pi(m_1,m_2)v - \big(1 - \pi(m_1,m_2)\big)\frac{\pi(m_1,m_2)v - a(m_1,m_2)}{1 - \pi(m_1,m_2)}.$$

Hence, following the structure of $a(m_1,m_2)$ given in Eq. (1) of the main text, we can express the cost function $c(m_1,m_2)$ as

$$c(m_1,m_2) = \frac{\pi(m_1,m_2)v - a(m_1,m_2)}{1 - \pi(m_1,m_2)}, \tag{SI.5}$$

from which

$$c(m,m) = \frac{\pi(m,m)v - a(m,m)}{1 - \pi(m,m)} = \frac{\frac{1}{2}v - a(m,m)}{\frac{1}{2}} = v - 2a(m,m).$$

Consequently, if ESS $\mathbf{p}^* = \mathbf{p}^*(m,m) = (p_1^*, 1 - p_1^*)$ is the ESS for a trait value m, then

$$p_1^* = p_1^*(m,m) = \frac{v}{v - 2a(m,m)} = \frac{v}{c(m,m)},$$

and so the product $p_1^*(m,m)c(m,m)$, that is, the per game average of the maximal possible cost of playing hawk in a monomorphic ESS population is always v independently of m.

SI.4

Examples of local extrema in $c(m,m)$ at symmetric ESP

In our example in the main text, both $c(m.m)$ and $a(m,m)$ are constant. Here, we show that this is not the general situation and a strict maximum or minimum can occur at $m = m^*$. To find such a function, we rotate the graph of function $a(m_1, m_2)$ defined by Eq. (7) of the main text around (m^*, m^*). If

$$u_\varphi(m_1, m_2) = m^* + \cos(\varphi)(m_1 - m^*) + \sin(\varphi)(m_2 - m^*)$$

and

$$w_\varphi(m_1, m_2) = m^* - \sin(\varphi)(m_1 - m^*) + \cos(\varphi)(m_2 - m^*),$$

then the graph of

$$a_\varphi(m_1, m_2) := a\big(u_\varphi(m_1, m_2), w_\varphi(m_1, m_2)\big)$$

is the graph of $a(m_1, m_2)$ after a rotation of angle φ around (m^*, m^*). Let us denote the corresponding cost function calculated according to (SI.5) by $c_\varphi(m_1, m_2)$.

For example, if $m^* = 5/8$ and

$$\varphi_1 = 30^\circ, \text{ then } u_{\varphi_1}(m_1, m_2) = \frac{\sqrt{3}}{2} m_1 + \frac{m_2}{2} + \frac{5(1-\sqrt{3})}{16}$$
$$\text{and } w_{\varphi_1}(m_1, m_2) = -\frac{m_1}{2} + \frac{\sqrt{3}}{2} m_2 + \frac{5(3-\sqrt{3})}{16},$$

and the cost $c_{\varphi_1}(m,m)$ is minimal, and so $a_{\varphi_1}(m,m)$ is maximal at $m = m^*$.

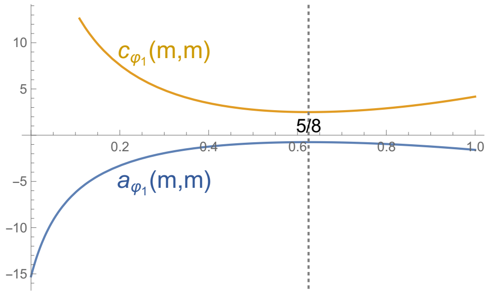

Fig. SI.1 The graphs of $c_{\varphi_1}(m,m)$ and $a_{\varphi_1}(m,m)$

While if

$$\varphi_2 = -30^\circ, \text{ then } u_{\varphi_2}(m_1, m_2) = \frac{\sqrt{3}}{2} m_1 - \frac{m_2}{2} + \frac{5(3-\sqrt{3})}{16}$$
$$\text{and } w_{\varphi_2}(m_1, m_2) = \frac{m_1}{2} + \frac{\sqrt{3}}{2} m_2 + \frac{5(1-\sqrt{3})}{16},$$

and the cost $c_{\varphi_2}(m,m)$ is maximal, and so $a_{\varphi_2}(m,m)$ is minimal at $m = m^*$.

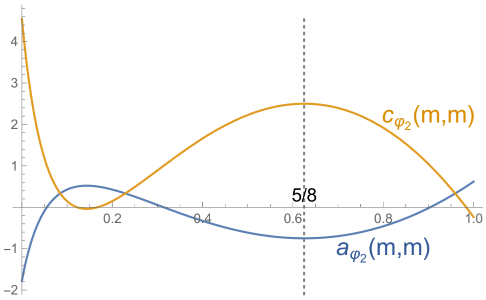

Fig. SI.2 The graphs of $c_{\varphi_2}(m, m)$ and $a_{\varphi_2}(m, m)$

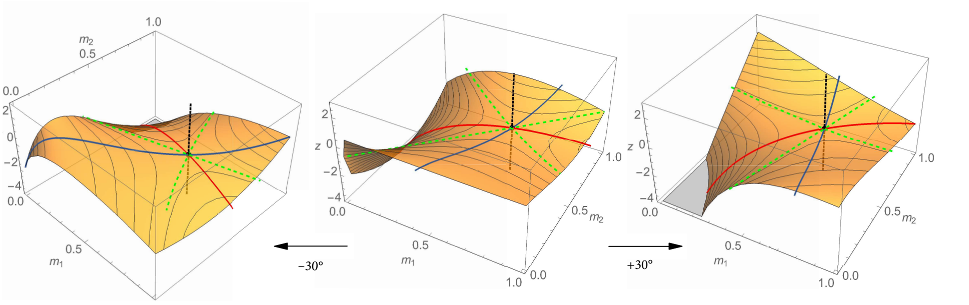

Fig. SI.3 The figure in the middle is the graph of the $a(m_1, m_2)$ defined by Eq. (7) of the main text. The figures on the left and the right show the graphs of the functions derived from $a(m_1, m_2)$ by rotating its graph by $-30°$ and $30°$ around the dashed black line, respectively. The dashed green curves make up the level line corresponding to the value $a(m^*, m^*)$, and their intersection is the point on the graph corresponding to (m^*, m^*). When the graph of $a(m_1, m_2)$ is rotated by $-30°$ $(+30°)$, then the blue (red) curve ends up above the points with (m, m) coordinates.
